# Evaluating the Added Value of Digital Contact Tracing Support Tools for Citizens: Framework Development

**DOI:** 10.2196/44728

**Published:** 2023-11-29

**Authors:** Ruth Baron, Nora Hamdiui, Yannick B Helms, Rik Crutzen, Hannelore M Götz, Mart L Stein

**Affiliations:** 1 Centre for Infectious Disease Control National Institute for Public Health and the Environment Bilthoven Netherlands; 2 Department of Health Promotion Care and Public Health Research Institute Maastricht University Maastricht Netherlands; 3 Department of Public Health Municipal Public Health Service Rotterdam-Rijnmond Rotterdam Netherlands; 4 Department of Public Health Erasmus MC University Medical Center Rotterdam Rotterdam Netherlands

**Keywords:** contact tracing, digital tools, citizen involvement, COVID-19, infectious disease outbreak, framework, mobile phone

## Abstract

**Background:**

The COVID-19 pandemic revealed that with high infection rates, health services conducting contact tracing (CT) could become overburdened, leading to limited or incomplete CT. Digital CT support (DCTS) tools are designed to mimic traditional CT, by transferring a part of or all the tasks of CT into the hands of citizens. Besides saving time for health services, these tools may help to increase the number of contacts retrieved during the contact identification process, quantity and quality of contact details, and speed of the contact notification process. The added value of DCTS tools for CT is currently unknown.

**Objective:**

To help determine whether DCTS tools could improve the effectiveness of CT, this study aims to develop a framework for the comprehensive assessment of these tools.

**Methods:**

A framework containing evaluation topics, research questions, accompanying study designs, and methods was developed based on consultations with CT experts from municipal public health services and national public health authorities, complemented with scientific literature.

**Results:**

These efforts resulted in a framework aiming to assist with the assessment of the following aspects of CT: speed; comprehensiveness; effectiveness with regard to contact notification; positive case detection; potential workload reduction of public health professionals; demographics related to adoption and reach; and user experiences of public health professionals, index cases, and contacts.

**Conclusions:**

This framework provides guidance for researchers and policy makers in designing their own evaluation studies, the findings of which can help determine how and the extent to which DCTS tools should be implemented as a CT strategy for future infectious disease outbreaks.

## Introduction

### Background

One of the most essential strategies to help mitigate the spread of infectious diseases is contact tracing (CT) [1], which was the driving strategy leading to the global eradication of smallpox and is still used to control the transmission of infectious diseases, such as the regularly resurging Ebola [2]. Studies conducted during the COVID-19 pandemic have also shown CT to be effective in preventing a substantial number of new infections [3,4]. CT traditionally consists of 3 phases: identification (first phase) and notification (second phase) of the contacts of individuals with COVID-19 and monitoring the health (third phase) of individuals with COVID-19 and their contacts. These tasks are generally conducted by public health services (PHSs) that are responsible for CT in many countries.

In 2020, the new SARS-CoV-2 coronavirus led to an unexpected global disruption at various levels, owing to the substantial morbidity and mortality it caused and the ease at which it spread. Although the Netherlands and many other Western countries were previously designated as being within the most prepared tier for handling epidemics and pandemics [5], the COVID-19 pandemic exposed weaknesses in the ability to contain the spread of the virus even within this tier. As has been reported in many countries worldwide, the high infection rates have caused CT for COVID-19 to be labor-intensive and time-consuming, and health services have been overwhelmed [6,7]. The potential of digital tools to rapidly access and transmit information to large numbers of individuals is being explored and used as ways to help contain the spread of the coronavirus and reduce the burden on overwhelmed health services [8,9].

### Proximity-Based Apps

Given the increased use of smartphones, many mobile apps have already been developed for detecting those who have been in close proximity to and possibly exposed to a person with an infection [9]. The first such well-known app for COVID-19 was developed in Singapore (TraceTogether) [10]. This app and many other apps that were subsequently developed, including the Dutch CoronaMelder [11,12], use Bluetooth, Wi-Fi, or GPS to detect individuals who have been in close proximity to those who have contracted COVID-19 within a critical time frame of infectiousness [13,14]. Positive effects of proximity-based apps in containing the virus have mainly been shown by modeling studies, and a few real-life studies have found some promising preliminary results when comparing Bluetooth-based and other proximity-based apps with traditional CT [15-17]. Using such apps may identify at least twice as many infected cases as traditional CT does and could potentially help to avert hundreds of thousands of positive cases owing to the feature of being able to notify individuals who are presymptomatic [18]. However, proximity-based apps require a high adoption rate by the general population (irrespective of whether they are infected with SARS-CoV-2) to be effective. Besides privacy concerns [19-21] and interoperability issues between countries [22,23], however, there are practical implications and expectations from citizens to be effective, such as needing to carry a smartphone wherever one goes, continually keeping Bluetooth on, and correctly following the instructions and abiding by the measures upon receiving a notification of having been in contact with a positive case. As the data from these proximity-based apps are generally not shared with health services conducting CT, these apps do not directly alleviate the burden of traditional CT experienced by public health professionals (PHPs). PHPs will still not have insight into the contacts that have been identified and who need to be informed and will therefore still need to conduct CT as usual when they have contact with positive cases (henceforth, referred to as index cases).

### Digital CT Support Tools

To complement traditional CT and proximity-based apps in helping to contain the virus, there are other types of tools that have been developed and are continuing to be developed. These tools are referred to as digital CT support (DCTS) tools and enable citizens to assume at least some of the tasks required for traditional CT. As has been reported with proximity-based apps, people may also have some privacy concerns, regarding the privacy of their own and their contacts’ personal data in the tool and what happens when these data are transmitted to the PHP conducting CT. When compared with traditional CT and proximity-based apps, DCTS tools offer unique advantages. They can alleviate the work of PHPs, by potentially shortening the time needed in traditional CT to collect contact details from them over the phone and to notify their contacts. Other possible benefits are more time for index cases to be able to recall more contacts they may have exposed and higher chance of recording more accurate contact details, compared with when sharing this information during a phone call with the PHP. Depending on the national CT measures being implemented, index cases may also notify and convey the correct instructions to their contacts more rapidly than if this responsibility is left to PHPs. A functionality in the tool that enables the index case to indicate to the PHP which contacts have already been notified would save PHPs time in having to reach the contacts themselves. DCTS tools that enable index cases and contacts to assess and monitor their own symptoms are already well known; in some cases, they can send these data to PHPs for further support, thereby contributing to the monitoring phase (third phase) of CT [24-26]. Much less attention has been given to DCTS tools that mimic the first 2 phases of CT: identification and notification of contacts.

During the COVID-19 pandemic DCTS tools were developed that had proximity-based and contact diary features where index cases themselves could actively collect their contacts and their contacts’ personal details [27]. Once this information had been gathered by the index case, they could choose to share it with a PHP. An app developed in the Netherlands (GGD Contact app), for example, had an interactive functionality enabling index cases to directly receive data from and share data with the municipal PHSs that are tasked with CT [28]. Data collected by the index case (eg, the test date and information about contacts) could be transmitted directly into the case management system used by the PHPs conducting CT, theoretically saving data entry time for the PHP. Another functionality in this app enabled the index case to retrieve contact details, such as telephone numbers, directly from the standard contact list already stored on their mobile phone.

When using such a DCTS tool during the first phase of CT, the index case recalls the contacts whom they may have exposed to the virus and enters information into the tool (eg, website or app) about the type of contact (eg, whether the contact is a partner or household member); nature of the meeting with the contact (eg, whether they were closer than 1.5 meters for >15 min); and finally, their contact details, such as telephone numbers and email addresses. The index case can then share this information with the PHPs conducting CT. During the second phase of CT, the index case or the PHP informs the contacts about their possible risk of infection and provides them with instructions about the steps to take to mitigate the further spread of infection (eg, to quarantine). Instructions may be provided in these DCTS tools customized to the type of contacts identified by the index case; these instructions can subsequently be shared by the index case with the relevant contacts. The index case can also use this DCTS tool to indicate which contacts they have notified and have provided with instructions. This information can also be shared with the PHP.

**Figure 1:**
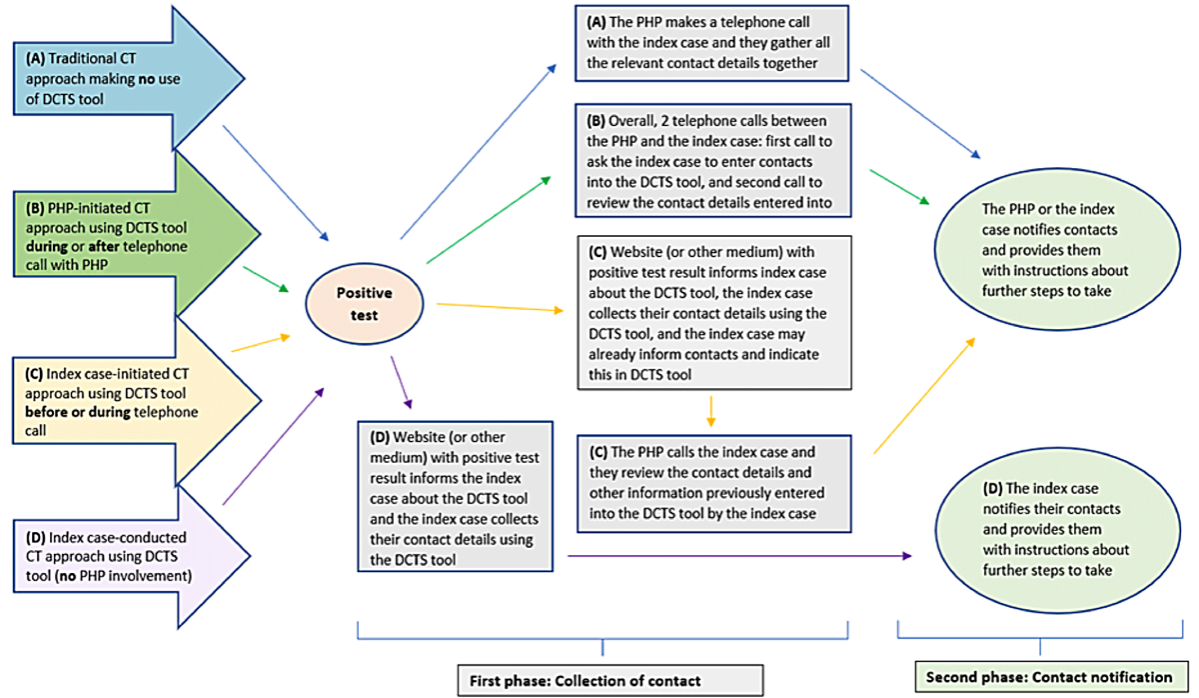
Flowchart showing 4 contact tracing (CT) approaches—(A) the traditional CT approach, (B) public health professional (PHP)-initiated CT approach making use of a digital CT support (DCTS)-tool, (C) index case-initiated CT approach making use of a DCTS tool, and (D) index case-conducted CT approach making use of a DCTS tool.

Besides the traditional CT approach (approach A in Figure 1), we distinguish 3 main approaches representing differing levels of citizen involvement that could potentially be taken with respect to DCTS tools for this process (approaches B to D in Figure 1). In traditional CT (approach A), the PHP plays the greatest role and the index case plays the smallest role. Going from approach B to approach C and finally approach D, the efforts of the index case in the CT process increase.

In approach B, using DCTS tools (PHP-initiated CT), the PHP calls the index case to start the CT process. Together, they complete a questionnaire to gather relevant demographic and medical details about the index case. The index case is then asked if they are willing to enter their relevant contact details into the DCTS tool, such as a designated website or app. The PHP and index case arrange a new appointment shortly afterward to review the contacts that have been provided by the index case, to determine whether additional information is needed and to agree on who will inform the contacts and provide them with instructions about further steps to take.

In approach C, using DCTS tools (index case–initiated CT) may be more efficient for CT (eg, alleviating the burden experienced by PHPs and increasing the speed of CT), as the index case is made aware of the DCTS tool before the call with the PHP during which the CT process is initiated. The designated medium to communicate test results to citizens (such as a website or email) can direct index cases to enter their contact details into the DCTS tool. This information is then ready to be discussed and shared digitally before the call with the PHP. This approach could lead to increased accuracy in contact details (such as telephone numbers), as these will not be conveyed verbally during the phone call. During the call, the provided information can be checked; additional details can be shared, if necessary; and further steps can be discussed.

In approach D, using DCTS tools (index case–conducted CT), the entire CT process is transferred into the hands of citizens or at least to a selection of citizens with low risk. In this situation, there is no contact at all between the PHP and the index case. The index case independently uses the DCTS tool to help recall and notify relevant contacts about their possible exposure risk and provides contacts with appropriate instructions already programmed in the tool. This could potentially free up time for PHPs to focus on index cases with greater health risks or with limited access to DCTS tools. This approach may be beneficial if the virus variant is very infectious (leading to high numbers of index cases) but relatively less severe with regard to its effects on morbidity and mortality.

Additional features of DCTS tools could facilitate the recording of events that the index case had attended or venues they had visited during the period that they were infectious, where they may have exposed groups of people. This recording of events could occur by various means, each varying in terms of privacy and ease of use, including digital tracking of attendees, scanning of a QR code by attendees, or self-reporting by index cases. Information regarding the event, such as its name, location, and address; phone numbers; time it occurred; and how many people were possibly exposed could be recorded. These details could be transmitted to the PHP conducting CT or directly to a representative of the event, so that they could alert the other attendees about their exposure risk.

Irrespective of the extent of PHP involvement, enabling the index cases to notify their contacts and provide them with instructions themselves may lead to an earlier awareness and more rapid action by contacts, compared with traditional CT, and ultimately mitigate the further spread of the virus. This type of technology requires more effort by citizens; however, its success will depend heavily on the ability and willingness of citizens to actively contribute in this manner, by mimicking the traditional CT process in a digital way.

It is currently unknown whether DCTS tools that encourage citizens to support the CT process (approaches B to D) could contribute to a more effective and efficient CT process; reduce the workload of PHPs; and ultimately, help to mitigate the spread of infectious diseases, such as COVID-19. It is therefore important that evaluations are performed to determine empirically the added value for public health with respect to the current COVID-19 pandemic and potential future infectious disease outbreaks.

Frameworks about how to evaluate proximity-based apps and the effectiveness of CT in general have been published already [29-31], but none, to the best of our knowledge, have been published about how to assess the effectiveness of DCTS tools. This paper presents a framework for how DCTS tools can be assessed when compared with traditional CT, with regard to various important factors that are necessary to curb the transmission of infectious diseases, such as increasing the speed and comprehensiveness of the CT process and relieving the burden on PHPs. This framework may be useful for researchers and policy makers to help determine the extent to which DCTS tools would have a positive impact on CT during future infectious disease outbreaks and to make evidence-based decisions regarding the implementation of these tools (eg, in conjunction with traditional CT and proximity-based apps).

## Methods

### Overview

The evaluation framework was developed by researchers from the National Institute for Public Health and the Environment (*Rijksinstituut voor Volksgezondheid en Milieu*; RIVM) in the Netherlands, in collaboration with municipal PHSs conducting CT. The idea for a framework for DCTS tools was partly based on an evaluation model that was developed for measuring the effectiveness of the Dutch proximity-based app [32]. A team of scientific experts from various disciplines including infectious disease control, epidemiology, health behavior, data privacy, and technical innovations was established to provide advice to the research team conducting the study. Evaluation topics and accompanying research questions were formulated to assess the potential effects of using the Dutch DCTS tool. These topics and their rationale were based on the experiences of PHPs and the CT indicators already developed by infectious disease experts at RIVM [33]. A previous concept of the framework was presented several times to various expert groups, including the Ministry of Health, Welfare, and Sport and medical nurses and physicians specialized in infectious diseases, who were working in the municipal PHSs. They provided feedback and suggestions leading to adjustments in the framework. The framework was further refined and developed into this research paper by reviewing the relevant international and national scientific literature on CT.

In this paper, the evaluation topics and research questions have been embedded within an outcome evaluation and a process evaluation, which are considered to be beneficial for evaluating the effectiveness, efficiency, validity, and usability of an intervention [34]. The research questions formulated under the *Outcome Evaluation* section are designed to measure the effects of the DCTS tools on CT, including the number of contacts identified and informed, speed at which this process occurred, accuracy and comprehensiveness of the information provided, number of positive cases detected among contacts, and actual time spent by PHPs on CT when these tools are used. The research questions formulated in the *Process Evaluation* section are designed to examine whether the DCTS tool was used as intended by index cases and PHPs and to expose any unintentional factors influencing its effectiveness. The process evaluation consists of examining the adoption rate of the tool; demographics of the users of the tool and those of their contacts; and user experiences of index cases, their contacts, and PHPs.

First, we discuss some legal, ethical, and technical issues to be considered before the evaluation study can be conducted. Next, we present the finally established framework containing the evaluation topics, research questions, and rationale behind each of them. In addition, we suggest and describe the potential study designs, methods, and measures that may be used to explore and answer the research questions (Multimedia Appendix 1). Finally, we discuss the implications of the framework for various types of future infectious diseases.

### Legal, Ethical, and Technical Feasibility

#### Overview

Before starting an evaluation process, it is important to explore the legal, ethical, and technical possibilities when it comes to collecting citizen data, transferring the data to a research data management system, and analyzing and storing the data. The use of technology in general is increasing rapidly, and increasing amounts of personal data are being shared globally to help organizations improve their services, reach more clients, and generate more income. This flow of data also comes with the risk of misuse and theft of personal information. Even during emergency situations leading to substantial morbidity and mortality, it is broadly considered important that any type of CT and the collection and storage of personal information is conducted in an ethically responsible and secure manner and that individuals’ data are protected [35]. Various countries and regions have legal frameworks in place for the handling of personal data, including the Personal Information and Electronic Documents Act in Canada, Health Insurance Portability and Accountability Act in the United States, and General Data Protection Regulation (GDPR) in Europe [36].

#### Legal Feasibility

If a digital intervention such as the DCTS tool is implemented, there should be transparency to the public about how their data will be used and if and how the data will be transmitted to a centralized CT system, protected from hacking, and stored safely [37]. There are established laws when it comes to citizens sharing their personal data. When index cases in the Netherlands download the DCTS tool, for example, they are required to indicate that they have read the terms and conditions, which include the use of their personal data. These state that the use of personal data is necessary in the interests of public health and that these data are handled in accordance with GDPR [38]. They also state that the data sent and received by the users are encrypted and conveyed via a secure server. Users are informed that they can send and receive data for up to 48 hours after the DCTS tool is activated. They are notified that their data will be deleted after 2 weeks from the web portal (that is linked to the centralized database). They are also informed that they are not obligated to provide personal information but that doing so will serve the process of CT.

#### Ethical Feasibility

In a similar manner to providing data for CT, there must be transparency and clear communication to individuals regarding which personal data will be used by researchers in the evaluation study, how their data will be transferred to a research database, how secure the database will be from hacking and misuse, how long the personal data will be stored before deletion, what data will be anonymized, and how the data will be used for evaluation purposes. There must be good collaboration with the health services collecting these data from index cases to ensure that all parties understand the purpose and relevance of conducting the evaluation study. Judicial advice must be sought regarding the ethics of obtaining limited personal data that are collected by health services for purposes other than CT, such as research, as stated in article 6 (4) of GDPR and how these data can best be anonymized. Morley et al [39], for example, have considered these aspects when it comes to implementing proximity-based apps, by developing guidelines consisting of 16 questions to be answered, to help ensure their ethical justification.

#### Technical Feasibility

In conducting evaluation studies, researchers will be dependent on the stability and consistency of the data that are obtained from the digital tool. However, technology is constantly changing; the tool they plan to evaluate may have changes to its algorithms, features removed or added, or updates made, or its implementation may even be discontinued [40]. It is therefore important that potential barriers are anticipated and that there are contingency plans in place during the writing of their research protocols. As the data collected for CT will pertain to potentially millions of people, it will be of practical importance to consider, before starting the evaluation, how this vast amount of data can best be transmitted, stored, organized, and analyzed for research purposes and who should be able to access the data. The advice of cybersecurity experts will be essential to ensure that the data provided by index cases are safe and protected. Ideally, the principles of the Privacy by Design approach should be adhered to, where privacy is assured by default within the design of IT and data storage systems, throughout each phase from data collection, transmission, storage, and elimination [41].

### Ethical Considerations

The development of this study framework did not involve human participants. This study is part of the larger project Strategic Program of the RIVM (SPR IZB2.0 S/070008), which was conducted in accordance with the Declaration of Helsinki—Ethical Principles for Medical Research involving Human Subjects. Ethical clearance was obtained from the Medical Ethics Review Committee of the University Medical Centre Utrecht. The Committee confirmed that the Medical Research Involving Human Subjects Act (WMO) does not apply to this project (reference 21/062).

## Results

### Framework and Rationale Behind Evaluation Topics

In this section, we have first presented the evaluation framework in Table 1. It contains an overview of the evaluation topics and research questions, divided into an outcome evaluation and a process evaluation. The rationale behind each of these evaluation topics and research questions has been discussed. Tables S1 and S2 in Multimedia Appendix 1 contain potential study designs, methods, and other considerations that may be helpful for examining the research questions.

**Table 1:** Outcome and process evaluation framework for comparing the use of digital contact tracing support (DCTS) tools (approaches B to D) with traditional contact tracing (CT; approach A)^a^.

Evaluation topics			Research questions
**Outcome evaluation**			
	Speed	What are the effects of DCTS tools (approaches B to D) when compared with traditional CT (approach A) regarding the length of time between the positive test result and having notified all relevant contacts about the further steps to take?	
	Comprehensiveness	What are the effects of DCTS tools (approaches B to D) when compared with traditional CT (approach A) regarding the number of contacts and the completeness of contact information provided by the index case?	
	Contact notification	What are the effects of DCTS tools (approaches B to D) when compared with traditional CT (approach A) regarding the proportion of contacts notified and provided with instructions?	
	Positive case detection	What are the effects of DCTS tools (approaches B to D) when compared with traditional CT (approach A) regarding the number (and proportion) of positive cases detected among identified contacts?	
	PHP^b^ workload	What are the effects of DCTS tools (approaches B to D) compared with traditional CT (approach A) when it comes to time, effort, and human resources?	
**Process evaluation**			
	Diverse demographic adoption and reach	How do demographic groups differ with regard to those who adopt DCTS tools (approaches B to D) and those who experience traditional CT (approach A) among (i) index cases and (ii) contacts who are reached by PHPs and by means of these tools?	
	Experiences of index cases	What are the experiences and views of index cases regarding DCTS tools (approaches B to D) when it comes to aspects such as their usability, effectiveness, and information security? Why do some index cases choose not to use these tools?	
	Experiences of contacts	What are the contacts’ views toward and experiences with receiving instructions from the index case? What CT approaches (A to D) do contacts prefer? To what extent do contacts adhere to the instructions?	
	Experiences of PHPs	What are the experiences and views of PHPs conducting CT with DCTS tools (approaches B to D) with respect to aspects such as their usability? What are PHPs’ views about leaving the CT process completely in the hands of index cases?	

^a^For each question, the various approaches to using this technology can be compared (Figure 1).

^b^PHP: public health professional.

### Outcome Evaluation: Evaluation Topics and Rationale Behind the Research Questions

#### Speed: Effectiveness of DCTS Tools in Terms of the Duration of the CT Process

Some infectious agents, such as the virus leading to COVID-19, can spread extremely quickly from one person to another, which makes it important that those at risk are identified as quickly as possible, preferably before symptoms occur, to prevent them from inadvertently spreading the virus to others. A systematic review and meta-analysis revealed that the average incubation periods for COVID-19 caused by the Alpha, Beta, Delta, and Omicron variants were 5, 4.50, 4.41, and 3.42 days, respectively [42]. Considering that one can be infectious from 2 days before symptoms appear, identifying the contacts of a person who has tested positive must be done as quickly as possible. A DCTS tool may enable the rapid retrieval of existing contact details stored in the standard contact list on a mobile phone or the index case can enter the contact details manually into a DCTS tool. As index cases can inform their contacts themselves and send them customized guidelines using the DCTS tool according to the type of contact, the contacts may be informed either before or shortly after the call with the PHP with the correct information. It is plausible that DCTS tools may, therefore, help to save time and lead to contacts testing themselves for COVID-19 immediately, seeking medical help, going into quarantine, or being aware of exposure to and possibly having acquired the virus. In contrast, index cases may be unable, hesitant, or unwilling to notify their contacts themselves, which would lead to contacts not being aware of their exposure on time and possibly spreading the virus to others. It is therefore important that the time between receiving the positive test result and having notified all relevant contacts is evaluated. The average duration of the CT process can be compared among those using the various DCTS tool approaches (approaches B to D) and those experiencing traditional CT (approach A).

#### Comprehensiveness: Effectiveness of DCTS Tools in Terms of the Number of Contacts Identified and the Completeness of the Information Provided

For CT to be effective, it is important that no exposed contacts or at least as few as possible are left unidentified or with missing contact details. The potential number of contacts identified will likely depend on the memories of index cases, their willingness to share contact details, availability of these contact details, and ease with which these details can be shared [43]. The average numbers of contacts shared by index cases will also possibly be influenced by public precautionary measures implemented at the time of the study, such as working from home or avoiding too many visitors. PHPs may be trained to ask index cases the right questions while conducting traditional CT to elicit as many contacts as possible. However, it is also plausible that the use of DCTS tools may buy more time for index cases to calmly jog their memories and remember everyone they have recently encountered. This may lead to them remembering more contacts than they would have with traditional CT and to retrieving more accurate contact details, such as telephone numbers and email addresses. It would be beneficial, therefore, to compare the number of contacts (with complete and accurate information) between those using DCTS tools (approaches B to D) and those undergoing the traditional CT process (approach A).

#### Contact Notification: Effectiveness of DCTS Tools in Terms of the Proportion of Contacts Notified and Provided With Instructions

Solely collecting a list of contacts and sufficient contact details will not ensure effective CT. Contacts also need to be notified and provided with instructions about what steps to take. Although there are contacts that will appreciate being notified about their possible exposure, index cases may have concerns about causing discomfort for them, by notifying them about the steps they need to take [44]. Having to quarantine can be associated with negative feelings, such as anxiety, loss of freedom, loneliness, anger, and boredom and can have a financial impact [45,46]; thus, conveying instructions to contacts who may not have been infected will not be an easy task. Index cases may also feel hesitance in notifying contacts themselves, owing to possible stigma and feelings of shame. In contrast, index cases may prefer notifying their contacts themselves, owing to hesitance in sharing personal contact details with the PHP. The more contacts that are notified and informed with instructions, the more likely the virus can be controlled. It is important, therefore, to evaluate DCTS tools (approaches B to D) by examining the number of contacts that are notified and provided with instructions, compared with traditional CT (approach A).

#### Positive Case Detection: Effectiveness of DCTS Tools in Terms of Detecting Positive Cases Among Contacts

The main aim of CT is to identify infected cases before they have the chance to transmit the virus to others. Measuring the ability of a CT strategy to identify the number of contacts who have contracted the virus is therefore important to determine how effective it is [47]. For the same reasons described previously, an increased number of contacts may or may not be notified earlier with the help of DCTS tools, compared with traditional CT. It is plausible that if more contacts are notified earlier, they will also take a self-test or be tested by the PHS immediately, and greater number of positive cases will be identified sooner. If the DCTS tool has such a functionality, it may enable the identification of contacts who become new index cases by matching identical identification numbers. It would be beneficial to examine whether DCTS tools (approaches B to D) have any value compared with traditional CT (approach A) with respect to the average number and proportion of identified contacts per index who have tested positive for the infectious agent, such as SARS-CoV-2.

#### PHP Workload: Effectiveness of DCTS Tools When It Comes to Time Spent by PHPs, Effort, and human resources

CT during the COVID-19 pandemic has required huge amounts of resources (in terms of staff, time, and required efforts) to keep up with cases and to conduct CT thoroughly [6]. The consequences can be that those conducting CT are overburdened and have less time to conduct CT comprehensively and for other health care tasks. In many countries, individuals from other professional fields, medical students, and volunteers have been trained to work as contact tracers to keep up with the number of infected cases [7]. It is unknown what the effects would be on the PHP workload if the index case, using a DCTS tool, takes over part of the process by identifying their own contacts, collecting contact details, and then informing the contacts themselves. Although there could be some work for PHPs, if they need to check whether contacts have indeed been notified and provided with the correct instructions, for example, in theory, the index case conducting most CT themselves could save time for PHPs and possibly lead to a more comprehensive and effective CT process. This could also reduce the need for more human resources and having to hire individuals from other fields with limited experience in CT and leave those who can conduct CT skillfully to handle the groups that have higher risk and are more vulnerable.

### Process Evaluation: Evaluation Topics and Rationale Behind the Research Questions

#### Diverse Demographic Adoption and Reach: Differences Between Demographic Groups of Index Cases and Contacts Across the Various CT Approaches

Ideally, DCTS tools will appeal to and be of use to all relevant segments of the population in terms of, for example, age, ethnicity, education, income, and health status. The use of digital technology, however, tends to be associated with and is easier to use for younger, more highly educated, wealthier, and healthier people [12,48,49]. There are some sociodemographic disparities when it comes to contracting the SARS-CoV-2 infection, degree of experiencing severe respiratory disease after contracting the virus, and mortality owing to the virus, all to the disadvantage of older people, ethnic minority groups, and those with lower incomes [50-52]. Although the Netherlands, for example, is a wealthy country with relatively little income inequality compared with many other countries, a study using national statistics data covering the early months of the pandemic (March 2020 to June 2020) found that those with the lowest incomes were twice as likely to die from COVID-19 as those with the highest incomes [53]. It will be beneficial to take all these sociodemographic factors into account when assessing the effectiveness of DCTS tools. It is also plausible that such a tool facilitating more citizen autonomy in CT could enable index cases to be a bridge to those in sociodemographic groups that are normally hard to reach when it comes to health care. In instances where traditional CT is being conducted alongside the use of DCTS tools, it is also possible that mainly low-risk index cases will conduct CT themselves with the DCTS tool, leaving more time for PHPs to focus on traditional CT with higher-risk groups. Regardless of the effects of the DCTS tool, it is advisable to examine whether DCTS tools can contribute to diminishing the health disparities caused by the infectious disease.

#### Experiences of Index Cases: Experiences and Views of Index Cases Regarding DCTS Tools When It Comes to Aspects Including Usability, Effectiveness, and Information Security

The success of CT by means of such DCTS tools will depend on the ability and willingness of index cases and PHPs to actively participate in and support the CT process. Supporting and motivating individuals to be involved will be key to making CT with DCTS tools effective. It will be of interest to examine the views of index cases toward becoming more involved in the CT process as a citizen and what they think about using DCTS tools to facilitate this process.

Traditionally, two main types of citizen involvement have been distinguished: (1) patient participation, in which a person contributes to their own health care, and (2) public participation, where a person contributes to the health care of others in the public domain [54]. The type of participation required for DCTS tools appears to have some elements of both types. Using this technology requires that index cases not only assist in their own care and those close to them but also contribute to protecting public health by actively helping to contain the infectious disease. Some benefits associated with citizen participation in health care are increased empowerment, improved patient experience, lower health costs, and decreased burden on and improved health care services. One review reported that although many people felt a collective responsibility to participate in CT for infectious diseases, including COVID-19, there was also some mistrust and concerns about their privacy [55]. This review concluded that individuals appeared to be motivated by contributing to public health in general but especially if there was some personal benefit to it.

Besides gaining further insight into individuals’ general feelings toward citizen involvement in CT, it will be important to examine the experiences and views toward using DCTS tools for this purpose. Such technology should ideally be considered user-friendly, useful, and sufficiently trustworthy for index cases to be willing to enter personal data and details pertaining to their contacts.

There are likely to be similar factors expressed by users of proximity apps that could also influence the adoption of DCTS tools, such as privacy and trust issues, perceived personal and social benefits, and perceived ease of use [56], but there may also be other experiences to explore that are unique to users of DCTS tools. To gain further understanding of these experiences, it is important to hear from both the users of the tools themselves and from those who are unable or have chosen not to use them.

#### Experiences of Contacts: Experiences and Views of Contacts Regarding the Various CT Approaches When It Comes to Receiving and Following Instructions

Involving citizens more in the CT process depends not only on the ability and willingness of index cases but also on the ability and willingness of contacts to be notified and to follow the instructions. Contacts may have questions about or disagree with the instructions or feel they cannot follow them and may need additional support from PHPs [57]. Contacts may also prefer being notified verbally instead of digitally or may not appreciate being notified by an index case instead of a PHP. A previous Dutch study conducted among PHPs before the COVID-19 pandemic showed that although PHPs recognized the potential of a digital system in which index cases could play a large role in identifying and informing their contacts, there was some concern that contacts may not be provided with the correct instructions, that the contacts would not take these instructions seriously if they did not come directly from a PHP, or that contacts may be left with unanswered questions and worries that could best be discussed with a PHP [58]. It is essential to examine whether the manner in which contacts receive instructions affects their feelings toward and acceptance of these instructions and their ability to follow them.

#### Experiences of PHPs: Experiences and Views of PHPs Regarding DCTS Tools, Including Their Usability and Transferring the CT Process Into the Hands of Citizens

As PHPs conducting CT have traditionally played the largest role in this process, their opinions about transferring all or a part of the CT process into the hands of citizens will be invaluable. They can be asked about their views regarding the willingness and ability of index cases to identify all relevant contacts and provide them with the correct information. According to a joint World Health Organization and European Centre for Disease Prevention and Control meeting report, countries anticipate that the CT of the future may require an approach that is more focused on groups that are vulnerable and at high risk [59]. PHPs may need to play an important role in determining which index cases can assume the CT role themselves with DCTS tools and which index cases would benefit more from traditional CT. Gaining some insight into how they will make these decisions, how they may encourage index cases to use the DCTS tool, and their views in general about involving citizens more in CT will be beneficial. It will also be important to examine their views regarding the usability (including user-friendliness) of DCTS tools; they can be asked about the features needed in DCTS tools to maximize their adoption by index cases. To provide context for and help to explain their views, it will be helpful to collect personal PHP characteristics. Their age and how long they have been conducting CT, for example, may influence their views in general about using new technology.

### Study Designs

To conduct the evaluation of DCTS tools, it is important to consider appropriate study designs and methods by which these study questions could be answered as optimally as possibly, despite the backdrop of a pandemic. The varying types of research questions will require a mix of study designs that need to be combined to answer all those questions. Some questions will require the quantitative examination of data collected from CT case management systems. These data may be obtained from centralized case management systems used by PHPs for CT or they may come from more decentralized databases collecting data from DCTS tools at a more local level, bypassing PHS. Other research questions will require the quantitative and qualitative assessments of information provided by users of DCTS tools themselves (index cases and PHPs) and their contacts, by means of surveys or interviews. In Tables S1 (outcome evaluation) and S2 (process evaluation) in Multimedia Appendix 1, study designs, methods, study measures, and relevant points of consideration are proposed, to answer each of the research questions outlined in the framework.

## Discussion

### Summary

The aim of this study was to develop and present a framework containing a set of research questions and accompanying study designs that may be valuable for a complete assessment of the identification and notification of contacts in CT using DCTS tools. Our framework is divided into an outcome and a process evaluation that assess the effects of DCTS tools, when compared with traditional CT. For the outcome evaluation, relevant topics to examine are the speed and comprehensiveness of the CT process, proportion of contacts who are notified and provided with instructions, proportion of contacts who test positive, and PHP workload (in terms of time, effort, and human resources). For the process evaluation, it is important to examine the demographics of those using the tools when compared with those who experience traditional CT and hear about the personal experiences of index cases, contacts, and PHPs when it comes to using these tools.

DCTS tools combine the advantages of digital technology with the ability and willingness of citizens to play a role in protecting their own health and that of others by assuming all or a part of the CT process themselves. The main added value of DCTS tools for CT of COVID-19 was that citizens could alleviate the work of PHPs when there were high numbers of cases. These types of tools could be of benefit to curb other future infectious disease outbreaks with high infection rates. For smaller-scale infectious disease outbreaks, particularly those with short incubation periods or those that are very infectious (requiring rapid CT), those leading to large numbers of contacts (requiring time, memory, and accuracy by the index case to collect contact details), and those associated with stigma (requiring more discrete or anonymous CT), DCTS tools could also potentially be of value [44]. Infectious diseases such as mpox (monkeypox) and sexually transmitted infections may have some stigma attached to them, forming a potential barrier to sharing contacts’ details with a PHP or notifying contacts [60-62]. DCTS tools can assist with partner identification and notification [63]. If index cases are uncomfortable with notifying contacts themselves or prefer to stay anonymous, contact details can be collected using the tool and shared with the PHP. The PHP can then reach out to the contacts and notify them, while enabling the index cases to remain anonymous. If index cases are concerned about sharing their contacts’ details with the PHP, contact notification could bypass the PHP, and the tool could facilitate the notification of and provision of instructions to contacts by the index cases themselves. If index cases prefer to stay anonymous, they could inform their contacts anonymously using an additional feature in the tool.

DCTS tools could theoretically be customized to any infectious disease for which CT is conducted, for example, by containing disease-specific information to help cases identify potential contacts who are at risk of exposure and through notification messages to advise contacts about further steps to take. DCTS tools may have added value when it comes to CT and curbing a wide range of future infectious disease outbreaks, and this framework can be used to measure the extent to which they are effective.

### Conclusions

This framework was developed to help researchers and policy makers design studies for their own evaluation of DCTS tools. The findings of these evaluation studies can provide guidance in determining how and the extent to which DCTS tools should be implemented as part of the CT strategy used for future infectious disease outbreaks.

## Appendix

Multimedia Appendix 1 Proposed study designs, methods and measures, as well as additional considerations for the outcome and process evaluations.

## Abbreviations

CT: contact tracing
DCTS: digital contact tracing support
GDPR: General Data Protection Regulation
PHP: public health professional
PHS: public health service
RIVM: Rijksinstituut voor Volksgezondheid en Milieu (National Institute for Public Health and the Environment)
