# Supplementary material for: Evaluating the Added Value of Digital Contact Tracing Support Tools for Citizens: Framework Development
Source: JMIR Res Protoc. 2023 Nov 29;12:e44728. doi: 10.2196/44728 (PMC10719815; doi:10.2196/44728)
Supplement: Multimedia Appendix 1 [file resprot_v12i1e44728_app1.doc]

**Multimedia appendix 1**

This is a multimedia appendix to a full manuscript published in the J Med Internet Res. For full copyright and citation information see doi:10.2196/44728.

**Table S1**. **Outcome evaluation**: proposed study designs, methods and measures, as well as additional considerations.

| **Research question (1)** |
| --- |
| What are the effects of DCTS-tools (approaches B-D) when compared to traditional CT (approach A) with regard to…  (a) the length of time between the positive test result and having notified all relevant contacts of further steps to take?  (b) the number of contacts and completeness of contact information provided by the index case?  (c) the proportion of contacts notified and provided with instructions?  (d) the number (and proportion) of positive cases detected among identified contacts? |
| **Study design and data collection** |
| *Study design:*  Longitudinal or cross-sectional study (depending on the way the data is registered and made available for research purposes, see below) comparing two (preferably similar) groups: one group undergoing the traditional CT process (approach A) without DCTS-tools and one group making use of DCTS-tools (approaches B-D). If multiple approaches with DCTS-tools are being implemented, the effects of these different approaches can also be compared.  *Data collection:*   - Routinely collected data by the PHS to conduct CT that is stored in case management systems could be made available for research purposes. Alternatively, data could be collected which bypass the centralized CT case management systems and is collected from more local CT case management systems or databases storing data obtained from DCTS-tools. - The aim of the first research question (1a) is to gain insight into the speed of various CT approaches. If digitally recorded events during CT are linked to timestamps, there will be multiple points of data within the same participant and the length of time between these events can be measured. It may be possible to have timestamps linked to the positive test result, as well as to the moment that all contacts have been collected and have been notified. This would be considered a *longitudinal* study. On the other hand, if timestamps accompanying relevant events are not available for research, it may be possible to have more aggregate data where measurements of time have been pre-calculated. In this case, there may be one time point of measurement for each participant and study would be *cross-sectional.* |
| **Study measures** |
| *Potential outcome measures:*   - Number of days/hours/minutes between the positive test result and the sharing of contact details with the PHS by the index case - Number of days/hours/minutes between the positive test result and having informed all the contacts - Number of and type of contacts that are identified per index case - Number and type of contacts per index case with complete information - Number and type of contacts informed and provided with instructions by the index case or PHP - Number and type of contacts with a positive test result per index case   *Potential confounders:*  Age, gender, education, migration background, native language, profession, area of residence, digital skills, health status, wariness and distrust toward sharing contact details, wariness and distrust toward technology in general.  Potential confounders should be examined and adjusted for in the analyses to help ensure that any effects of the DCTS-tools can be attributed to the technology itself, instead of to the users of such technology. Older people, for example, may be less inclined to use DCTS-tools and it is possible that CT already takes longer with them than with younger people, which makes age an important potential confounder that could mask the true effects of DCTS-tools. |
| **Additional considerations** |
| - To be able to assess the effects of the DCTS-tool approaches compared to the traditional tool approach, these approaches are ideally being implemented during the same timeframe, but in different locations. If traditional CT and the use of DCTS-tools are used in the same location, there is a chance that there may be large differences between the groups, which may be hard to control for, such as demographic, health and attitude differences. There would need to be sufficient low risk ‘healthy’ index cases in both groups, for example, to enable a more objective comparison. - It is possible that public health services are implementing one or more of the DCTS-tool approaches (B-D). Whatever is being implemented by public health services at that moment can be used to compare with traditional CT, or with other DCTS-tool approaches. All comparisons will give valuable information about the effects of varying levels of index case involvement on CT. - Data entered into CT case management systems by PHPs as well as data entered into the DCTS-tool by index cases will need to be made available for researchers, in an anonymous manner. It will be necessary to implement technical and ethical procedures to ensure the complete, correct and anonymous flow of data to be evaluated. - Important background variables that should ideally be the same or similar for all the CT approaches (A-D) that are being examined are the following: - season of the year (as these can be associated with infection rates) - time since the start of the pandemic - infection rate - virus variants - current national guidelines to control the virus spread - lockdown status - vaccination rates - This same study can also be used to for the process evaluation where the index case demographics associated with the CT approaches are examined (see research question 4). |
| **Research question (2)** |
| **(2)** What are the effects of using DCTS-tools (approaches B-D) compared to traditional CT (approach A) when it comes to time, effort and manpower? |
| **Study design and data collection** |
| *Study design:*   - Longitudinal or cross-sectional survey - Qualitative study with PHPs.   *Data collection:*   - Obtaining different measurements in time to show trends in perception within participants as the infection rate or other factors change will be beneficial. If following the same PHPs over time is unfeasible, conducting multiple cross-sectional surveys with different PHPs will provide valuable findings. Earlier assessments may help to raise issues which could be addressed quickly. Later assessments are useful to determine how well the DCTS-tool has been implemented and integrated within the CT process, and its effect on the PHP workload. - The qualitative study could consist of face-to-face (real-life or online) interviews, to obtain a deeper understanding of their views toward DCTS-tools when it comes to reducing their workload. - These studies will ideally be completed by a diverse range of PHPs from different regions where CT is conducted. PHPs will need to be asked for their informed consent before participating. |
| **Study measures** |
| Potential outcome measures and discussion topics for the qualitative study:   - Time and effort spent on telephone calls and administrative tasks - Number of contacts identified per index case in PHP’s view - Length of time for CT process per index case - The effect on necessary manpower for CT   For analyses examining differences in PHP characteristics with regard to their beliefs and views, collecting the following variables will  be useful.  *Independent variables (PHP characteristics)*   - Medical role - Length of experience with CT - length of experience with CT for COVID-19 - Duration of training for CT - Age |
| **Additional considerations** |
| - Data that are not possible to obtain from the more objective DCTS-tool and CT case management system (e.g. pertaining   to the speed of the CT process and the accuracy of the data) could be obtained by asking PHPs themselves in this study.   - These studies for the outcome evaluation can also be used for the process evaluation (see research question 6), where PHP are asked about the usability (including user-friendliness) of the DCTS-tool. |

**Table S2. Process Evaluation: proposed study designs, methods and measures, as well as additional considerations.**

| **Research question (3)** |
| --- |
| **(3)** How do demographic groups differ with regard to those who adopt DCTS-tools (approaches B-D), and those who undergo traditional CT (approach A) among:  (a) index cases?  (b) contacts who are reached by the PHS and by means of these tools? |
| **Study design and data collection** |
| *Study design:* Cross-sectional study  *Data collection:*   - To answer these questions, researchers can make use of the same data obtained from the PHS case management system for research question 1. Unlike question 1, it is not necessary to follow the participant to obtain the length of any period, but to make use of variables that indicate the type of CT approach that the index case is following. - PHS case management systems may only be able to share limited demographic data pertaining to index cases and their contacts, due to the ethical requirements for anonymity. A smaller-scale survey where index cases give their informed consent may offer possibilities for examining the association with demographics. Index cases would be able to provide information regarding the CT approach they prefer, as well as the demographics of the contacts they notified. |
| **Study measures** |
| Statistical analyses could be conducted comparing DCTS-tool users with those undergoing traditional CT with respect to demographics.  *Main outcome measure:*   - Type of CT approach (approaches A-D)   *Independent variables* (if available):   - Age - Gender - Education - Profession - Migration background - Native language - Area of residence - Demographic characteristics of contacts   *Demographic variables of contacts:*  The second cross-sectional study design where index cases complete a survey, would be suitable for gaining insight into associations between demographic groups of index cases and their contacts. Index cases could be asked about the demographics (such as age, gender, education and migration background) of the contacts they identified, notified and provided with instructions. |
| **Additional considerations** |
| - It would be of interest to repeat this cross-sectional survey over time as DCTS-tools increasingly become embedded within the traditional CT process, to determine if and how the associations between demographic groups and the CT approaches they follow, change. - All CT approaches (A-D) and their associations with demographic groups can be examined and compared. - The second smaller-scale cross-sectional survey (not making use of PHS data) can also be used for the analysis of research question 4. |
| **Research question (4)** |
| **(4)** Index cases: What are the experiences and views of index cases using DCTS-tools (approaches B-D) regarding aspects, such as their usability, effectiveness and information security? How do using DCTS-tools (approaches B-D) compare with traditional CT (approach A)? |
| **Study design and collection** |
| *Study design:*   - Cross-sectional study - Qualitative study   *Data collection:*   - The cross-sectional survey mentioned in research question 3, which is used to obtain information about the association of demographics with type of CT approach, can also be used to obtain answers to this research question. - Qualitative analysis based on face-to-face interviews (real-life or online) can be held with index cases of diverse background characteristics to obtain more insight into their experiences with using the various DCTS-approaches of these tools (B-D). - For both studies, index cases will be required to give their informed consent to participate. |
| **Study measures** |
| Outcome measures for the cross-sectional study and potential discussion topics for the qualitative study:  *Attitudes* *toward:*   - The COVID-19 virus and the corresponding public precautionary measures - CT and the PHPs who carry out CT - Contributing towards the health of others and mitigating the spread of the infectious agent - Trying out new technologies   *Opinions regarding:*   - The user-friendliness and ease of using DCTS-tools - The ability of index cases to collect contact data and inform contacts with DCTS-tools - The trustworthiness of DCTS-tools - Information security - Sharing contact details with PHPs for CT - Their feeling about others finding out the index case has the infectious disease   These outcome measures could also be treated as *independent variables* to determine any relationship with the following outcome measure:   - Intention to use DCTS-tools   Demographic characteristics of the index cases can be examined as *independent variables* to determine their association with these attitudes, opinions and intentions. |
| **Additional considerations** |
| - It will be important to recruit individuals who are recent index cases who have fresh memories of CT and who have used or are using DCTS-tools. - The index case could be invited to complete the cross-sectional survey by any medium that informs them of their test-result, such as a website or e-mail. A link can be provided to an online version of the survey. |
| **Research question (5)** |
| **(5)** What are contacts’ views toward and experiences with receiving instructions from the index case? What CT approaches (A-D) do contacts prefer? To what extent do contacts adhere to the instructions? |
| **Study design and data collection** |
| *Study design*:   - Cross-sectional study - Qualitative study   *Data collection:*   - The cross-sectional survey would ideally be completed by recent contacts who have been - contacted by index cases using DCTS-tools. - The qualitative study could consist of face-to-face interviews (real-life or online) with - contacts with diverse background characteristics. - For both studies, contacts will be required to give their informed consent to participate. |
| **Study measures** |
| *Outcome measures* for the cross-sectional study and potential discussion topics for the qualitative study:   - Proportion of contacts notified by the different CT approaches (A-D) - Preferences of CT regarding different CT approaches (A-D) - Proportion of contacts reporting adherence to instructions - Actions implemented as a result of received instructions - Intention to use DCTS-tools themselves   *Opinions regarding*:   - Being notified of possible exposure digitally and by an index case - Instructions being provided by index cases versus PHPs - Willingness to adhere to instructions and reasons for adherence (or lack of adherence) - Reasons for their preferences for certain CT approaches (A-D) |
| **Additional considerations** |
| - As with the recruitment of index cases, it will be important that the participants are recent contacts, so that their memories of being contacted are fresh. They will also need to have been notified by index cases who are using DCTS-tools. - To recruit contacts, index cases who were surveyed and interviewed could be asked to provide details of their contacts who are willing to participate. There could also be a general invitation for individuals who were recent contacts to participate in the study. The second option may encourage more objective and open responses. |
| **Research question (6)** |
| **(6)** What are the experiences of PHPs conducting contract tracing with DCTS-tools (approaches B-D) with respect to aspects such as their usability? What are PHPs’ views of leaving the CT process completely in the hands of index cases? |
| **Study design and data collection** |
| *Study design****:***   - Cross-sectional study - Qualitative study   *Data collection:*  The same cross-sectional survey and face-face interviews described earlier for research question 2 can be utilized for these research questions. |
| **Study measures** |
| *Outcome measures* for cross-sectional study and potential discussion topics for the qualitative study:   - Usability (e.g. user-friendliness) of using DCTS-tools and ideas for improvement - The ability of index cases in general to use DCTS-tools and to assume part of the CT process themselves - The willingness of index cases to use DCTS-tools for collecting and sharing contact details - The degree to which the correct information will reach contacts if the index case assumes this part of CT - The potential need for support of PHPs by contacts - The persuasive arguments (if any) a PHP may use to encourage the index case to use DCTS-tools - The reasons why a PHP would or would not recommend DCTS-tools to an index case (e.g. too old, too sick, too few contacts, etc)   For the cross-sectional study, as in research question 2, demographic differences between PHPs regarding the usability of DCTS-tools could be examined, with regard to these views. |
| **Additional considerations** |
| - The cross-sectional survey and interviews with PHPs described in research question 2, could be used to obtain information for the process evaluation. This question focuses less on the PHPs’ perspective of actual effects on CT and the workload for the PHP, but on the usability of the DCTS-tool for index cases. - The PHP may play a role in whether or not the index case makes use of the DCTS-tool. This question therefore aims to gain insight into what factors would encourage the PHP to introduce or prevent the PHP from recommending the DCTS-tool to the index case. |
